# Co-culturing with *Streptococcus anginosus* alters *Staphylococcus aureus* transcriptome when exposed to tonsillar cells

**DOI:** 10.3389/fcimb.2024.1326730

**Published:** 2024-01-25

**Authors:** Srijana Bastakoti, Maiju Pesonen, Clement Ajayi, Kjersti Julin, Jukka Corander, Mona Johannessen, Anne-Merethe Hanssen

**Affiliations:** ^1^ Department of Medical Biology, Research group for Host-Microbe Interaction (HMI), UiT – The Arctic University of Norway, Tromsø, Norway; ^2^ Oslo Centre of Biostatistics and Epidemiology, Oslo University Hospital, Oslo, Norway; ^3^ Department of Biostatistics, Institute of Basic Medical Sciences, Faculty of Medicine, University of Oslo, Oslo, Norway; ^4^ Parasites and Microbes, Wellcome Sanger Institute, Cambridgeshire, United Kingdom; ^5^ Helsinki Institute of Information Technology, Department of Mathematics and Statistics, University of Helsinki, Helsinki, Finland

**Keywords:** co-culture, *Staphylococcus aureus*, transcriptome, tonsillar cells, throat colonization

## Abstract

**Introduction:**

Improved understanding of *Staphylococcus aureus* throat colonization in the presence of other co-existing microbes is important for mapping *S. aureus* adaptation to the human throat, and recurrence of infection. Here, we explore the responses triggered by the encounter between two common throat bacteria, *S. aureus* and *Streptococcus anginosus*, to identify genes in *S. aureus* that are important for colonization in the presence of human tonsillar epithelial cells and *S. anginosus*, and further compare this transcriptome with the genes expressed in *S. aureus* as only bacterium.

**Methods:**

We performed an *in vitro* co-culture experiment followed by RNA sequencing to identify interaction-induced transcriptional alterations and differentially expressed genes (DEGs), followed by gene enrichment analysis.

**Results and discussion:**

A total of 332 and 279 significantly differentially expressed genes with p-value < 0.05 and log_2_ FoldChange (log_2_FC) ≥ |2| were identified in *S. aureus* after 1 h and 3 h co-culturing, respectively. Alterations in expression of various *S. aureus* survival factors were observed when co-cultured with *S. anginosus* and tonsillar cells. The serine-aspartate repeat-containing protein D (*sdrD*) involved in adhesion, was for example highly upregulated in *S. aureus* during co-culturing with *S. anginosus* compared to *S. aureus* grown in the absence of *S. anginosus*, especially at 3 h. Several virulence genes encoding secreted proteins were also highly upregulated only when *S. aureus* was co-cultured with *S. anginosus* and tonsillar cells, and iron does not appear to be a limiting factor in this environment. These findings may be useful for the development of interventions against *S. aureus* throat colonization and could be further investigated to decipher the roles of the identified genes in the host immune response in context of a throat commensal landscape.

## 1 Introduction

The influence of bacterial composition and interactions in the throat during frequent recurrence of staphylococcal colonization and infection are poorly understood. The anterior nares are considered the primary site of *Staphylococcus aureus* colonization (Wertheim et al., 2005; Hanssen et al., 2017) but several studies in healthy individuals indicate that *S. aureus* pharyngeal or throat carriage may be equally, or even more common (Ringberg et al., 2006; Mertz et al., 2007; Hamdan-Partida et al., 2010; Hamdan et al., 2018; Erikstrup et al., 2019). Indeed, the prevalence of *S. aureus* has been shown to be significantly higher in the throat (45%) than in the nose (40%) (Erikstrup et al., 2019), and the throat is considered to represent an important reservoir for methicillin-resistant *S. aureus* (MRSA) (Ringberg et al., 2006; Ide et al., 2009; Jang et al., 2014).

Aside from *S. aureus*, other aerobic and anaerobic opportunistic pathogens have also been found to colonize the throat regions, such as alpha and beta-hemolytic streptococci (group A, C, G), *Haemophilus influenzae, Haemophilus parainfluenzae, Enterococcus spp, Klebsiella pneumoniae, Corynebacterium* spp.*, Peptostreptococcus, Fusobacteria, Bacteroides and Veillonella* (Dickinson et al., 2020; Katkowska et al., 2020; Buname et al., 2021). The *Streptococcus anginosus* group (SAG), is the most common beta-hemolytic group C streptococci isolated from the human throat (Al-Charrakh et al., 2011). *S. anginosus* is a part of the normal human flora, commonly colonizing tonsils, and the upper respiratory, gastrointestinal, and reproductive tracts (Jiang et al., 2020; Buname et al., 2021). *S. anginosus* can cause dental abscesses and is associated with pharyngitis and tonsilitis (Shinzato and Saito, 1994; Mukae et al., 2016).

The interaction between *S. aureus* and *S. anginosus* is not well understood and, the influence in the growth and metabolism of both species during throat colonization is unclear. A bacterial co-culture can induce interspecies competition for nutrients, space, and attachment sites in their environment, and eventually enhances antibiotic resistance and virulence (Pajon et al., 2023). For instance, an interaction study between *S. aureus* and *Pseudomonas aeruginosa* indicates expression of virulence factors that can reduce metabolism in *S. aureus* through multiple mechanisms (Pastar et al., 2013; Nguyen and Oglesby-Sherrouse, 2016; Noto et al., 2017) and ultimately result in an enhanced virulence capacity and increased antibiotic tolerance (DeLeon et al., 2014). *S. aureus* can enhance virulence of *P. aeruginosa* through the release and assimilation of peptidoglycan component N-acetyl glucosamine (GlcNAc) (Korgaonkar et al., 2013; Yang H. et al., 2020). Another study has also shown the promotion of *S. aureus* colonization of lung tissue in the presence of *P. aeruginosa* due to upregulation of cell receptors in the lung tissue, which are absent in *S. aureus* infection alone (Millette et al., 2019).

During colonization of the respiratory tract, pathogens compete with pre-existing commensal bacteria. Bacteria adapted to particular hosts appear to be more capable of displacing a host’s microbiota (Iwase et al., 2010; Siegel and Weiser, 2015). The Interaction between two commensals can benefit colonization and increase the survival of both species in a specific site of the body (Jenkinson et al., 1990). This interaction can enable bacteria to adhere to host cells and can even increase their resistance to the host’s innate immune system (Asam and Spellerberg, 2014). Colonization with *S. aureus* constitute a significant risk factor for recurrent episodes of disease e.g., rhinosinusitis (Plouin-Gaudon et al., 2006), tonsillitis (Zautner et al., 2010) and osteomyelitis (Ellington et al., 2003) after the successful adhesion and invasion of the host cell. The success of *S. aureus* depends not only on adhesins and/or virulence genes and the ability to escape antibiotic treatment, but also on the coordinated and timely expression of genes upon infection of its host (Xu et al., 2016), which may change in the presence of another microbe. We have previously identified differentially expressed key determinants in *S. aureus* in the presence of primary human tonsillar epithelial cell using RNA sequencing and pathway analysis (Bastakoti et al., 2023). In the present study, we aimed to identify differentially expressed genes (DEGs) in *S. aureus* when co-cultured with *S. anginosus* in the presence or absence of a tonsillar cell line using the same experimental set up. This allowed us to observe an alteration in the gene expression landscape in *S. aureus* during co-culture with another frequent throat colonizer and compare the DEGs results between the present and our previous study.

## 2 Materials and methods

### 2.1 Experimental design

**Figure 1:**
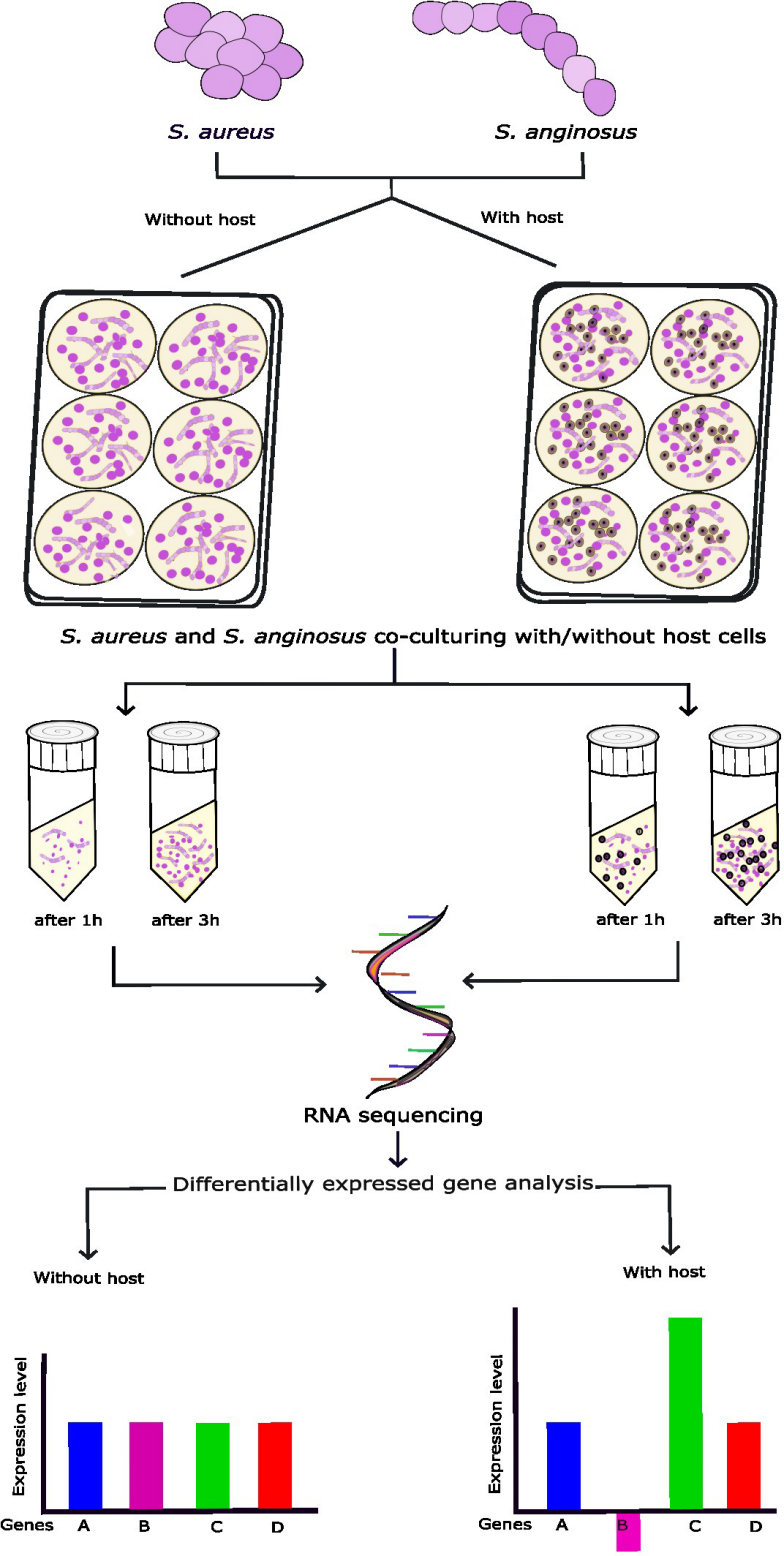
Schematic representation of the *in vitro* co-culturing of *S. aureus* and *S. anginosus* with or without tonsillar cells. *S. aureus* and *S. anginosus* were added to poly-L-lysin (PLL)-coated wells containing host media without tonsillar cells or with the presence of monolayer of host cells and incubated for 1h or 3h. Three independent experiments were run in triplicates. The adhered bacteria were collected for RNA extraction. The RNA samples were further processed for RNA-seq followed by differentially expressed genes (DEGs) analysis.

The experimental setup is schematically illustrated in **Figure 1**. In this study, a *Staphylococcus aureus* throat isolate was co-cultured with *Streptococcus anginosus* and a tonsillar cell line to study the alteration in the transcriptome in *S. aureus*.

### 2.2 Handling of cells

Human Tonsil Epithelial Cells (HTEpiC, Cat #2560, Sciencell, United States) used in this study were handled and sub-cultured according to the manufacturer´s instructions (Sciencell Research Laboratories, California). The tonsillar cells were grown in poly-L-lysin (PLL) coated flask. Briefly, tonsillar cells were incubated at 37°C in a 5% CO_2_ incubator together with Tonsil Epithelial Cell Medium (TEpiCM), 1% Tonsil Epithelial Cell Growth Supplement (TEpiCGS) and penicillin/streptomycin solution (P/S).

A *Staphylococcus aureus* strain TR145 (SAMEA112465883) was isolated from a healthy human throat (Sangvik et al., 2011) and was studied for the first-time concerning co-culturing. The *Streptococcus anginosus* (ATCC 33397) strain originated from human throat tissue, it is ß-hemolytic and typed as Lancefield’s group G (Bauer et al., 2020; Kuryłek et al., 2022). *S. anginosus* was grown according to the handling information provided by ATCC and *S. aureus* was grown as described previously (Bastakoti et al., 2023). Briefly, bacterial cultures were incubated separately in Brain Heart Infusion (BHI) media overnight at 37°C with shaking at 220 revolutions per minute (rpm). At first, both bacteria were grown on BHI overnight at 37°C, and then the fresh bacterial culture was prepared (1:10) for monoculture and co-culturing (*S. aureus* grown in the presence of *S. anginosus*) to get an overview of the bacterial growth pattern for up to 3 hours (h).

### 2.3 *In-vitro* co-culturing of *S. aureus* with *S. anginosus* and tonsillar cells

HTEpiC was cultured until passage four and seeded at a density of ~ 4 × 10^5^ viable cells per well in six-well plates or ~ 7 × 10^4^ viable cells per well in 24 well plates coated with poly-L-lysin (PLL). The HTEpiC was grown until confluence, washed with Dulbecco’s Phosphate-Buffered Saline (DPBS, Sciencell, Cat #SC0303) and added Tonsil Epithelial Cell Medium (TEpiCM) before being exposed to bacteria.

At first, both the bacterial strains, *S. aureus* and *S. anginosus*, were grown to log phase OD of ~ 1 and adjusted to OD of ~ 0.4 (corresponding to ~ 1 x 10^8^ CFU/ml). The separate bacterial inoculum was prepared with Multiplicity of Infection (MOI) of 5. The co-culture experiment was conducted in 6 well plates with 2 ml working volume: a 1 ml *S. aureus* inoculum followed by 1 ml of *S. anginosus* inoculum was added into the well seeded with HTEpiC. Two different time points (1 h and 3 h) were selected for triplicate *in-vitro* experiments in 6-well plates. Following incubation at 37°C in the presence of 5% CO_2_ with tonsillar cell medium (TEpiCM), bacteria in the presence or absence of host cells were collected and total RNA was subjected to RNA-seq according to a previously described protocol (Bastakoti et al., 2023). Briefly, all unbound co-cultured bacteria were washed away, host cells were trypsinized and only those bacteria which had managed to attach to tonsillar cells were collected and processed for RNA-seq. As a control, only those bacteria that managed to attach to the PLL-coated wells containing host media (TEpiCM, no tonsillar cells) were collected and processed for RNA-seq. Additionally, *S. aureus* and *S. anginosus* monocultures with/without tonsillar cells were performed in parallel for colony forming units (CFU) plate enumeration.

To confirm the CFUs from co-culture, serial dilution of the collected bacteria was performed, followed by plate enumeration in selective strep agar (COBA medium) (cat no. #C754532, ThomasScientifc, USA) to detect *S. anginosus*, and CHROMagar plate to detect *S. aureus*, before incubation for 24 h at 37°C The plating was done in triplicate, and the average CFU/ml was calculated. For better understanding of host stress levels during the exposure time points, cytotoxicity assays were performed on co-culturing plates, as described earlier (Bastakoti et al., 2023).

### 2.4 NGS library construction and RNA sequencing

The RNA extraction was performed according to a previously described protocol (Bastakoti et al., 2023) following the recommendations from the manufacturer (RNeasy Mini Kit, Cat. No. 74104). Briefly, all samples were lysed enzymatically using lysozyme and lysostaphin, as well as mechanically disrupted using a homogenizer (Precellys Evolution, Bertin technologies) before RNA extraction followed by DNase treatment. Total RNA extracted from three replicates of *S. aureus* co-cultured with *S. anginosus* in the absence/presence of host cells collected at the time point of 1 h and 3 h, were processed for RNA-seq library preparation, as described previously (Bastakoti et al., 2023), using Lexogen’s CORALL™ Total RNA-Seq Kit with RiboCop (Cat.No.96; EU, CH, USA). No prior RNA fragmentation was needed in this protocol. The samples were sequenced on an Illumina 550 platform, with dual indexes, and paired end (PE) mode. The final sequencing concentration was 1.8 pM. The expected fragment length for PE reads was < 100 nucleotides.

### 2.5 Read quality control, trimming and mapping

**Figure 2:**
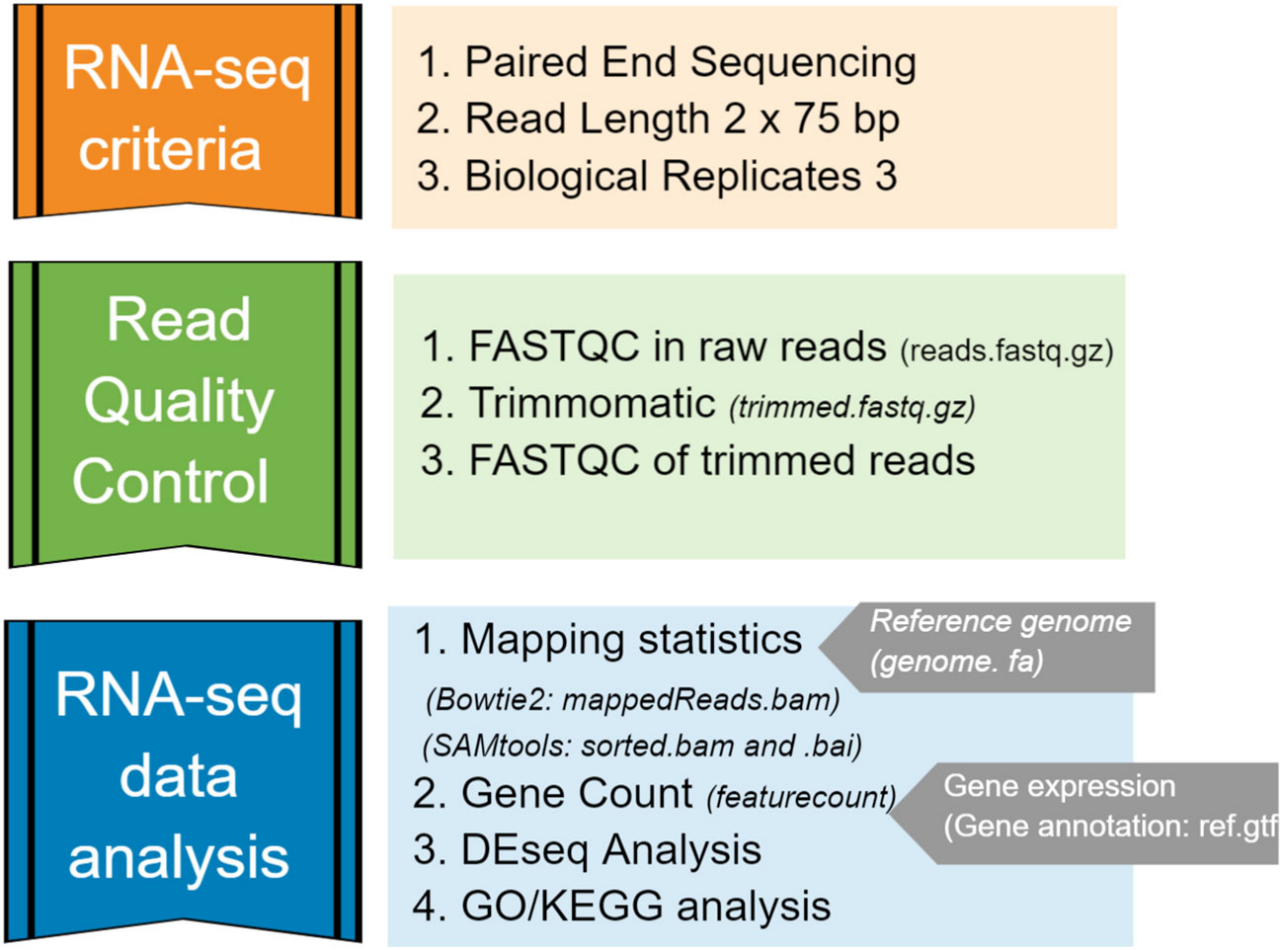
Bioinformatics pipeline used for RNA-seq data analysis in this study.

The overall pipeline for RNA-seq data analysis included generating FASTQ-format files containing reads sequenced from a next-generation sequencing (NGS) platform, quality control and trimming, aligning reads to an annotated reference genome, and quantifying expression of genes (**Figure 2**). Each library produced between 45 – 184 million reads, and they were pre-processed for a quality check using FASTQC/0.11.9-Java-11. Filtering (removing adaptor dimer reads) and trimming (removing low-quality bases) were performed by Trimmomatic/0.39-Java-11. Then, only those sequences with a quality score Q > equal to 20 and a minimum of 55 nucleotide sequence length were retained in the dataset. The final quality check was performed in the trimmed file.

Strain *S. aureus* TR145 (SAMEA112465883) and *S. anginosus* (ATCC 33397) were used as two separate reference genomes for the mapping performed by Bowtie2/2.4.4-GCC-10.3.0. Clean and trimmed RNA-seq reads were first mapped to the *S. aureus* genome, and then again to *S. anginosus*, in order to determine how many reads mapped to each bacterium. The percentage of mapping efficacy was retrieved from Bowtie2 and sorted by Samtools/1.14-GCC-11.2.0. Further, the gene count matrix for gene expression analysis was identified using the featureCounts program implemented in the SourceForge Subread package (Liao et al., 2013).

### 2.6 Differentially expressed gene analysis

Differentially expressed genes (DEGs) of the test group (*S. aureus* and *S. anginosus* with host cell) versus the control group (*S. aureus* and *S. anginosus* without host cell) were analyzed using the DESeq R package (1.38.0). Each group has three biological replicates per condition. DESeq2 count data were transformed using the variance stabilizing transformation (VST) for negative binomial data distributions with dispersion-mean trends (Anders and Huber, 2010). DEGs were calculated under log_2_ FoldChange (log_2_FC) ≥ |2| and a false discovery rate (FDR) adjusted *p* (p_adj_) < 0.05. The p values were adjusted using the Benjamini and Hochberg approach for controlling FDR. Genes with an adjusted p value less than 0.05 found by DESeq were assigned as differentially expressed. Any gene that followed this threshold was considered a good starting point for identifying significantly expressed genes. The DEGs exhibited by *S. aureus* from co-culturing with *S. anginosus* were then compared to *S. aureus* alone from our previous study (Bastakoti et al., 2023) to identify alteration in DEGs due to co-culturing with *S. anginosus* in the presence of tonsillar cells. An overview of total DEGs analyses and comparisons performed is shown in **Table 1**.

**Table 1:**
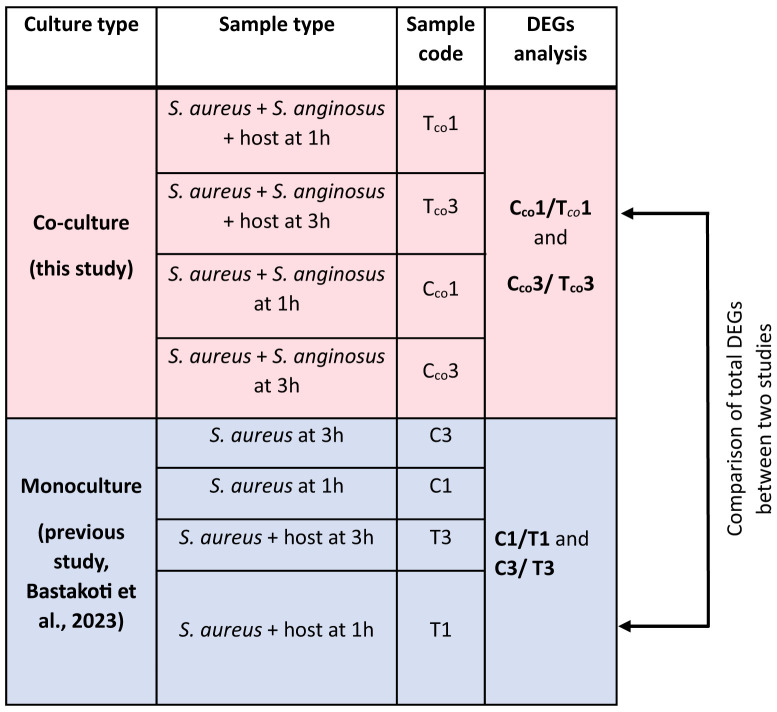
An overview of total DEGs analyses and DEGs comparisons performed in this study.

The red color shading represents sample type and RNA-seq data generated from the present co-culture study. The blue color shading represents sample type and RNA-seq data generated from the previous transcriptomics analysis of *S. aureus*.

### 2.7 GO/KEGG pathways enrichment analysis of DEGs

Gene Ontology (GO) enrichment analysis of the DEGs was performed by the ShinyGO 0.77 online software (http://bioinformatics.sdstate.edu/go/), in which pathways were sorted by fold enrichment. GO terms with a corrected FDR value of less than 0.05 were considered significantly enriched by DEGs. Pathway enrichment was determined using the Kyoto Encyclopedia of Genes and Genomes (KEGG) pathways annotation. String Database (v 11.5) (https://string-db.org/) was used to test the statistical enrichment of DEGs in the KEGG pathways. Pathways were considered significantly enriched with an FDR of less than 0.05.

## 3 Results

### 3.1 Recovery of bound bacteria in the presence and absence of tonsillar cells

Both bacterial strains, *S. aureus and S. anginosus*, were originally isolated from the human throat and were chosen as representative throat bacteria in this study. As we aimed to analyze *S. aureus* transcriptome altered due to presence of *S. anginosus* in the tonsillar cells, *in-vitro* infection assay was performed, and only surface-bound bacterial strains were collected and plated.

**Figure 3:**
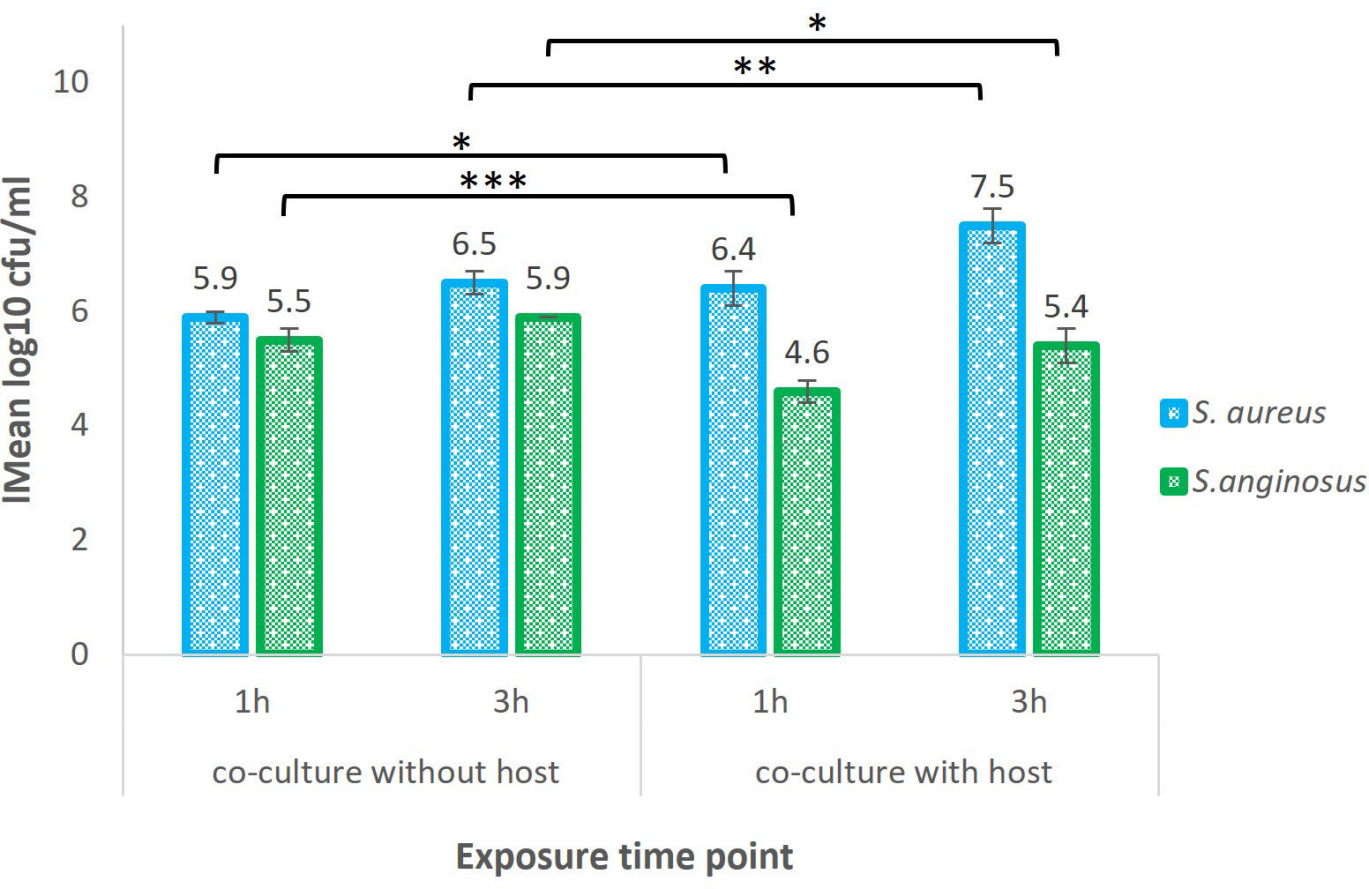
Recovery of *S. aureus and S. anginosus* co-cultured with or without tonsillar cells. S. aureus and S. anginosus were grown for 1 or 3 hours (h) in tonsillar cell medium, with or without tonsillar cells (host) at 37°C in the presence of 5% CO_2_. All unbound bacteria were washed away, and remaining bacteria were collected for RNA-seq and plated on selected media for CFU enumeration. The results are presented as mean log 10 CFU/ml from three independent experiments. Error bars represent the +/- SD. Differences in the means between the groups were tested using a two-sample Student t-test. *P < 0.05, **P < 0.01, ***P < 0.001.

The recovery of bound *S. aureus* when co-cultured with *S. anginosus* with host cells (referred to as test samples) was evaluated after 1 and 3 h and is presented in **Figure 3**. After 1 h, 6.4 log10 CFU/ml (corresponding to ~ 1.1 x 10^6^ CFU/ml) of bound *S. aureus* was recovered when co-cultured with *S. anginosus* with host cells, and after 3 h it was 7.5 log10 CFU/ml (corresponding to ~ 3.9 x 10^7^ CFU/ml). Whereas recovery of *S. aureus* co-cultured with *S. anginosus* in the absence of tonsillar cells (referred to as control samples) after 1 and 3 h was 5.9 log10 CFU/ml and 6.5 log10 CFU/ml, respectively. Likewise, the recovery of bound *S. anginosus* when co-cultured with *S. aureus* with host cells (referred to as test samples) was 4.60 log10 CFU/ml (corresponding to ~ 4.1 x 10^4^ CFU/ml) at 1 h and 5.4 log10 CFU/ml (corresponding to ~ 2.7 x 10^5^ CFU/ml) at 3 h (**Figure 3**). Whereas recovery of *S. anginosus* co-cultured with *S. aureus* without host cells (referred to as control samples) after 1 and 3 h was 5.5 log10 CFU/ml and 5.9 log10 CFU/ml, respectively (**Figure 3**). For the evaluation of the bacterial effect on tonsillar cell viability during *in-vitro* infection assay, the lactase dehydrogenase (LDH) release by the tonsillar cells was measured. The LDH release was found to be less than 5% (**Supplementary Figure S1**), indicating that the host cells remained viable during the co-incubation with *S. aureus* and *S. anginosus*.

Similar procedure was executed to identify the recovery of *S. aureus* during co-culturing with *S. anginosus* in the absence of host cell i.e., with host cell media (TEpiCM) in PLL-coated wells. A significant increase in the recovery of bound bacteria was observed for both *S. aureus* and *S. anginosus* from their individual growth when compared to recovery of both bacteria from co-culture set-up (**Supplementary Figure S2**). Taken together, despite a significant decrease in recovery of *S. aureus* when co-cultured with *S. anginosus* without a host cell, a significant increase in *S. aureus* recovery was identified when co-cultured with *S. anginosus* and host cells. The number of surface bound bacteria increased over time, and the recovery of *S. aureus* and *S. anginosus* indicated that all the samples could be proceeded for RNA seq.

Additionally, fresh bacterial culture prepared for *S. aureus* and *S. anginosus* in BHI was performed to visualize their growth pattern when grown alone and together, prior to *in-vitro* infection assay. The OD_600nm_ measured in 30-minute intervals for up to 3 h for alone and mix of both species, including the CFU/ml identified after 1 h and 3 h, are presented in **Supplementary Figure S3**. The results showed that the two species can co-exist when grown in BHI, especially at the time point chosen for infection assay.

### 3.2 Reads assigned to the *S. aureus* genome

RNA-seq was performed to study the transcriptome of *S. aureus* when exposed to *S. anginosus* with/without tonsillar cells. Total reads per library ranged from 45 – 184 million, with 44 - 183 million reads per library remaining after processing of the raw data.

The trimmed reads were aligned with the reference genome, and the mapping efficacies against the *S. aureus* reference genome ranged between 20 - 73% for all 1 h samples and between 55 - 69% for 3 h samples (**Table 2**). The detailed mapping efficacy for *S. aureus and S. anginosus* reference genome is presented in **Supplementary Table S1**. Overall, this finding revealed that most sequencing reads were mapped to the *S. aureus* reference genome, which corresponds to higher recovery of bound *S. aureus* compared to *S. anginosus.*

**Table 2:** Mapping efficacy of reads assigned against *S. aureus* genome.

Biological Replicates (BR)	Sample ID	Experimental conditions	Mapping efficacy (%)
**1st**	S1.ctr_1h.BR1	*S. aureus* + *S. anginosus*, control, 1h	73
	S2.trt_1h.BR1	*S. aureus* + *S. anginosus* + tonsillar cells, test, 1h	<20
	S3.ctr_3h.BR1	*S. aureus* + *S. anginosus*, control, 3h	64
	S4.trt_3h.BR1	*S. aureus* + *S. anginosus* + tonsillar cells, test, 3h	69
**2^nd^**	S5.ctr_1h.BR2	*S. aureus* + *S. anginosus*, control, 1h	64
	S6.trt_1h.BR2	*S. aureus* + *S. anginosus* + tonsillar cells, test 1h	47
	S7.ctr_3h.BR2	*S. aureus* + *S. anginosus*, control, 3h	63
	S8.trt_3h.BR2	*S. aureus* + *S. anginosus* + tonsillar cells, test, 3h	69
**3^rd^**	S9.ctr_1h.BR3	*S. aureus* + *S. anginosus*, control, 1h	66
	S10.trt_1h.BR3	*S. aureus* + *S. anginosus* + tonsillar cells, test, 1h	26
	S11.ctr_3h.BR3	*S. aureus* + *S. anginosus*, control, 3h	55
	S12.trt_3h.BR3	*S. aureus* + *S. anginosus* + tonsillar cells, test, 3h	61

### 3.3 Normalization of the gene read counts

**Figure 4:**
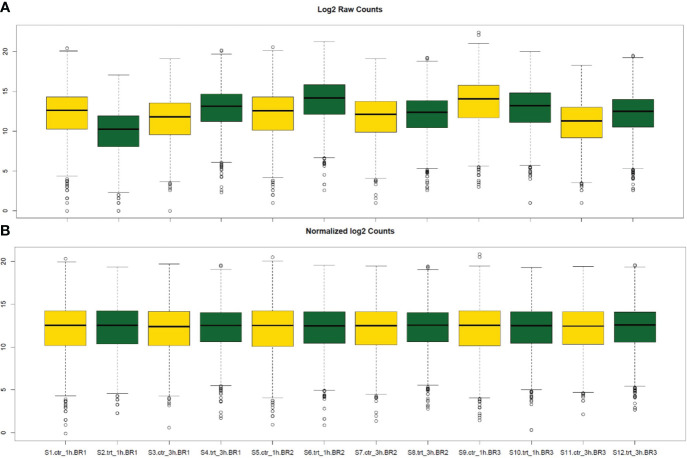
Box plot representation of raw read counts before and after DESeq2 normalization. The yellow and green box plots represent the *S. aureus* co-cultured with *S. anginosus* in the presence or absence of tonsillar cells, respectively (either at 1h or 3h). **(A)** The distribution of log2 raw counts before normalization. **(B)** The counts after DESeq2 normalization.

The normalization of read counts was used for gene count comparisons between *S. aureus* co-cultured with *S. anginosus* in the presence or absence of tonsillar cells. The representation of raw counts from all 12 samples (S1 to S12) is shown in **Figure 4A**. DEseq2 normalization has corrected variations in sequencing depths and biological replicates (**Figure 4B**). The results obtained after the normalization of gene counts are more reliable and accurate than raw counts, and thus normalized gene read counts are implemented for DEGs analysis.

### 3.4 Visualization of sample-to-sample distances in PCA plot

**Figure 5:**
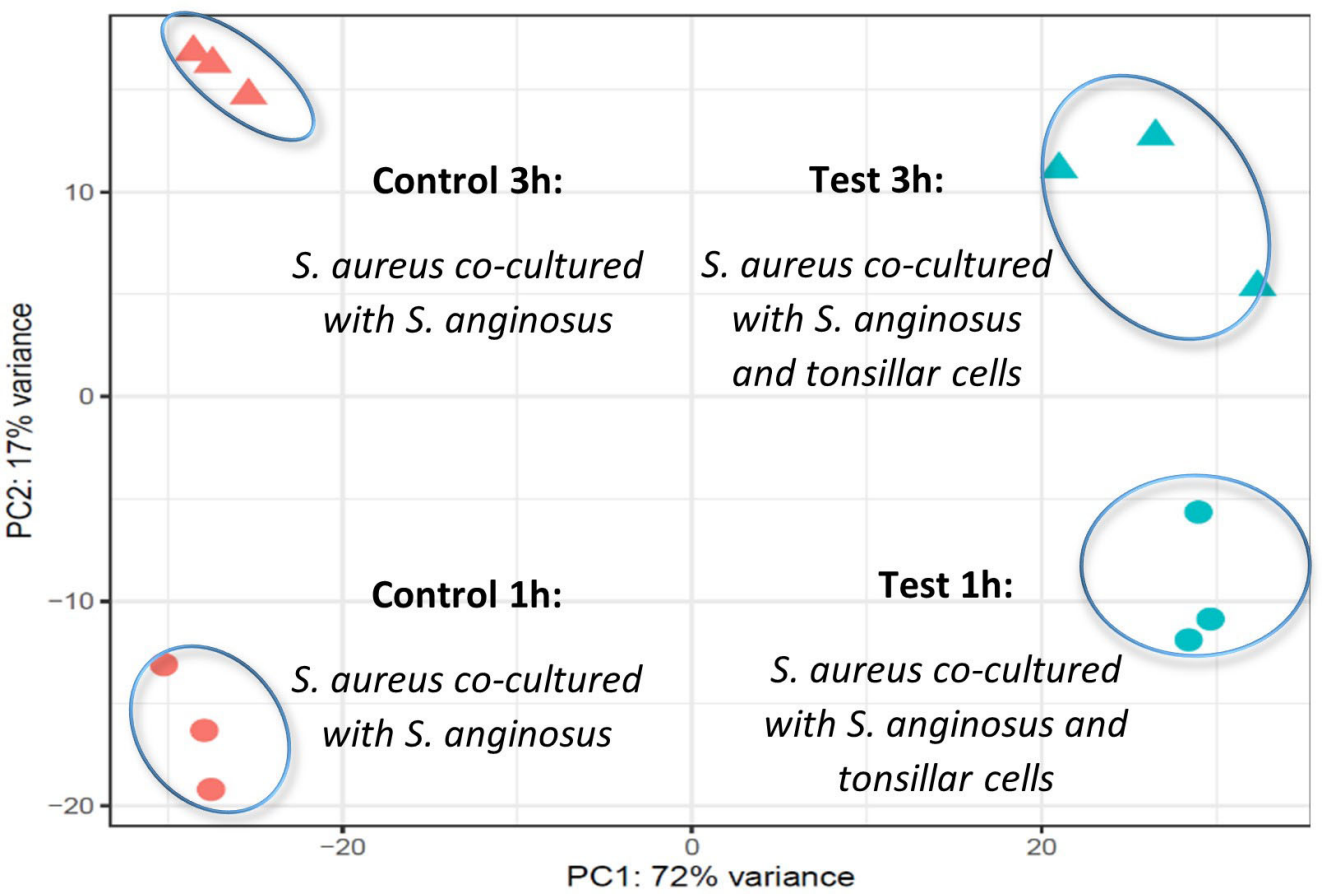
PCA plot using the variance stabilizing transformation (VST) values of the read counts. The circles and triangles represent 1 and 3 hours, respectively, while orange and blue color represent *S. aureus* co-cultured with *S. anginosus* without host cells (control) and *S. aureus* co-cultured with *S. anginosus* with host cells (test), respectively.

To visualize the between-group and between-time sample variance in the samples, a principal component analysis (PCA) plot was done before performing DEGs analysis. The PCA plot showed a clear clustering of RNA reads from the three biological replicas of *S. aureus* co-cultured with *S. anginosus* at 1 h or 3 h in the presence or absence of host cells (**Figure 5**). The difference between the two time points was considerable (PC2 explaining 17% of the overall variability), though not stronger than the differences due to exposure to host cells (PC1 explaining 72% of the overall variability). Hence, the results suggest that exposure to host cells induces more variability than temporal change.

### 3.5 Highest variance across sample and gene clustering

**Figure 6:**
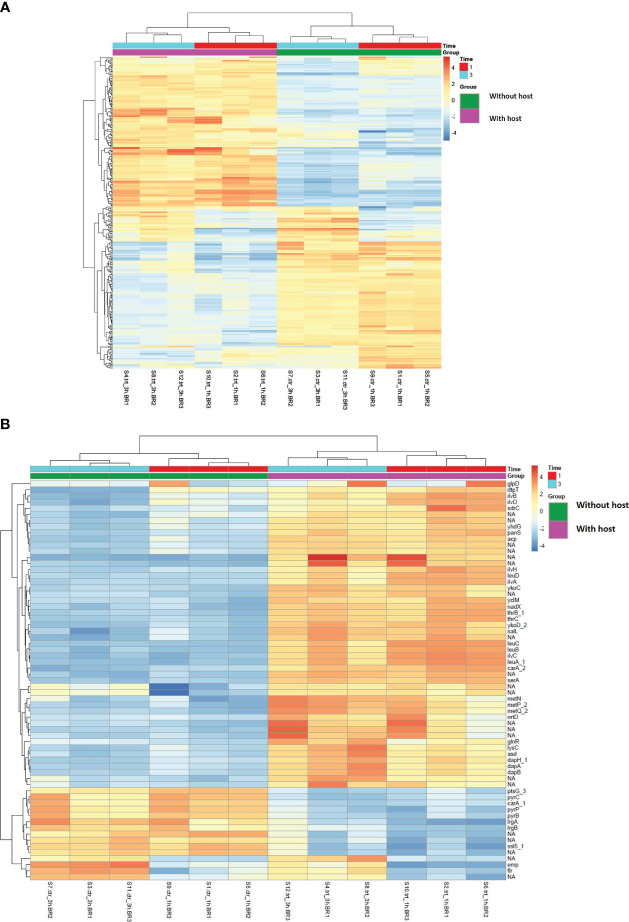
Heatmap showing gene clustering-based presence/absence of host cells for 1 and 3 hours in *S. aureus* using variance stabilizing transformation data. The values in the heatmap present mean-centered read counts after applying variance stabilizing transformation (VST). The gene clusters (pink and green) represent the gene clustering based on sample type i.e., in the absence and presence of tonsillar cells, while the different exposure times are represented in red (1h) and turquoise (3h). Blue-toned color indicates lower than average variation across samples, whereas orange-toned color indicates higher than average variation. **(A)** Clustering of the top 300 genes with the highest variation across the various conditions. **(B)** Clustering of the top 65 genes with the highest variation across the various conditions. Unannotated genes are marked with NA.

The overall gene clustering pattern of genes present in *S. aureus* co-cultured with *S. anginosus* in the absence (without host) and the presence of tonsillar cells (with host) is visualized in heatmaps (**Figure 6**). The 300 genes with the highest variation across samples are presented in **Figure 6A**. For a subset of genes (in the middle of the heatmap), the main source of variation was caused by the length of incubation (1 or 3 h). For a more detailed visualization of gene clustering, the top 65 genes with the highest variation across samples are presented in **Figure 6B**. It indicated two clear clusters of genes: the top 50 genes constructed one cluster where genes such as *glpD, dtpT, ilvB* and *sdrC* showed high variance in samples exposed to host cells, whereas the remaining 15 genes constructed the second separate cluster where genes such as *pyrC, irgA, emp* and *flr* showed high variance in samples with no exposure to host cells. Overall, the *S. aureus* transcript pattern is highly influenced by the co-culturing with *S. anginosus* in the presence/absence of the tonsillar cells.

### 3.6 DEGs in *S. aureus* after co-culturing

**Figure 7:**
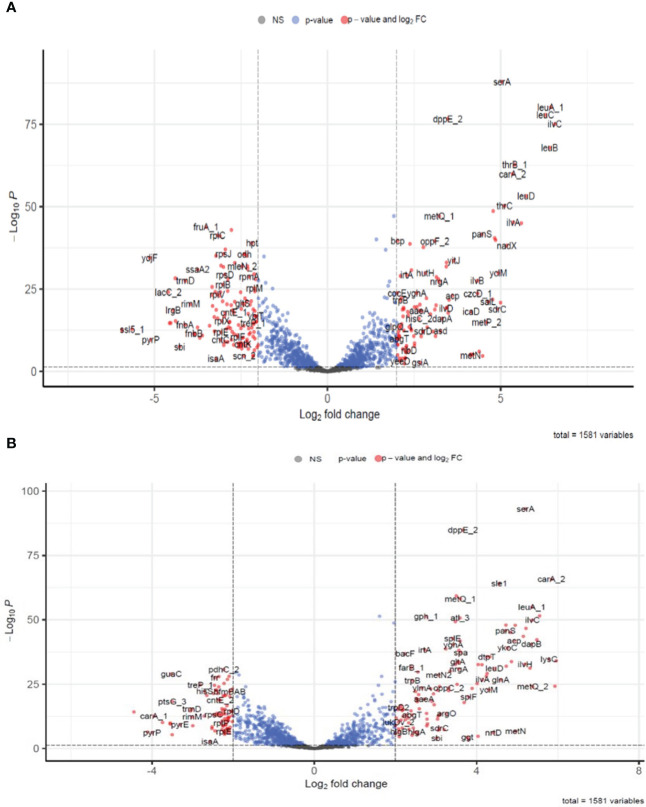
Differentially expressed genes identified in *S. aureus* co-cultured with *S. anginosus* and tonsillar cells at 1h and 3h. Only annotated genes were included in the plot. Blue dots represent genes with only significant p-value <0.05, red dots represent significantly differentially expressed genes (DEGs) with cut-off values with p-value < 0.05 and log_2_ fold change cut-off >2 and grey are non-significantly differentially expressed genes. **(A)** Significantly DEGs after 1h. **(B)** Significantly DEGs after 3h.

To identify the differentially expressed genes (DEGs) present in *S. aureus* co-cultured with *S. anginosus* in the presence of tonsillar cells compared to *S. aureus* co-cultured with *S. anginosus* in the absence of tonsillar cells, the normalized gene reads counts were analyzed using DESeq2. A total of 332 (at 1 h) and 279 (at 3 h) significant DEGs in *S. aureus* co-cultured with *S. anginosus* in the presence of host in comparison to *S. aureus* co-cultured with *S. anginosus* in the absence of host were identified. There were 242 commonly shared DEGs, of which 155 were annotated. The volcano plots in **Figure 7** represent annotated DEGs in *S. aureus* co-cultured with *S. anginosus* in the presence of tonsillar cells, i.e., 245 out of 332 DEGs at 1 h (**Figure 7A**) and 207 out of 279 DEGs at 3 h (**Figure 7B**). The total number of DEGs, excluding unknown genes, was 297.

Among the significantly upregulated genes with the highest log_2_FC at 1 h were *ilvC, leuA_1, leuB, leuC, leuD, ilvH*, *thrB_1, ilvA*, and *carA_2*, and among the highly downregulated genes were *lrgA, ssl5_1, ydjF, pyrP, sbi* and *fnbA* (**Supplementary Table S2**). Similarly, at 3 h, the top upregulated genes were *salL, metp_2, carA_2, lysC, ykoD_2, dapA, metQ_2, ilvC, leuA_1, dapB*, and *asd*, and while the top downregulated were *pyrC, lrgA, pyrP*, and *carA_1* (**Supplementary Table S3**).

*S. aureus* and *S. anginosus* genes may have some sequence similarity. Therefore, we evaluated whether the genes that were differentially expressed in *S. aureus*, could be found in S*. anginosus*. Of the 297 obtained DEGs, 185 genes were exclusively present in *S. aureus*. The remaining 112 DEGs could be a result of expression of both *S. aureus* and *S. anginosus* genes. These genes are listed in **Supplementary Table S4**. All DEGs were analyzed for functional enrichment analysis to identify any possible significant pathways involved by those genes in S. *aureus* during co-culturing.

In summary, the transcriptome of *S. aureus* revealed significant changes during co-culturing with *S. anginosus* upon exposure to tonsillar cell lines. Several transcripts were found to be unique to the tested time points, while others were expressed at both time points.

### 3.7 GO term enrichment analysis

**Figure 8:**
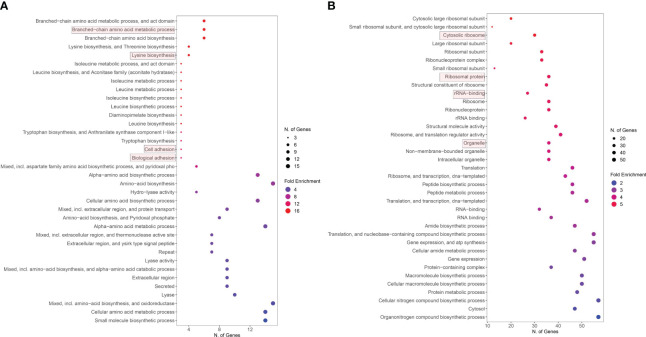
Enriched GO functions of DEGs. **(A)** Upregulated genes GO analysis. **(B)** Downregulated genes GO analysis.

All upregulated and downregulated genes derived from RNA-seq data analysis were separately applied in gene ontology (GO) enrichment analysis to identify enriched GO terms involved during *S. aureus* co-cultured with *S. anginosus* and tonsillar cells compared to *S. aureus* co-cultured with *S. anginosus* but without tonsillar cells. The GO enrichment analysis selected by FDR and sorted by fold enrichment revealed that significantly enriched upregulated DEGs were mainly involved in biological processes, including the “amino acid metabolic process”, “lysin biosynthesis”, “cell adhesion” and “biological adhesion” (**Figure 8A**). The downregulated DEGs were mainly enriched in “cytosolic ribosome”, “ribosomal protein”, “rRNA binding”, and “organelle” (**Figure 8B**).

**Figure 9:**
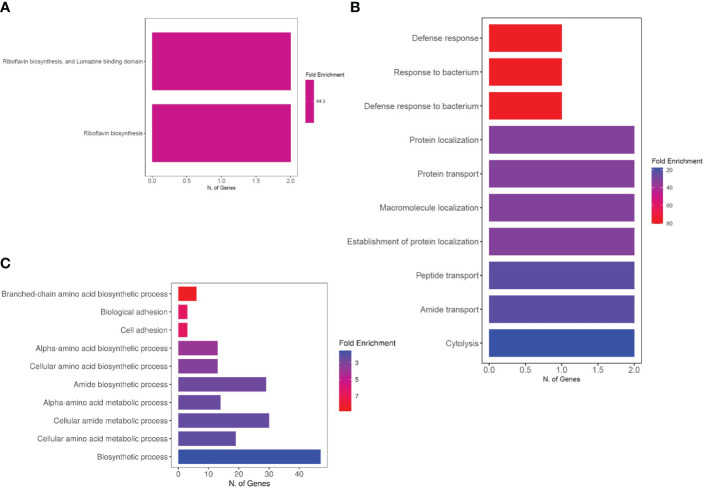
Enriched biological processes of DEGs. **(A)** Two biological processes identified from uniquely upregulated genes from 1 h of co-culturing. **(B)** Top 10 biological processes identified from uniquely upregulated genes from 3 h of co-culturing. **(C)** Top 10 biological processes identified from common DEGs after 1h and 3 h of co-culturing.

Additionally, the uniquely upregulated DEGs in co-cultured *S. aureus* with *S. anginosus* and tonsillar cells compared to *S. aureus* co-cultured with *S. anginosus* but no tonsillar cells, at 1 h were enriched in “riboflavin biosynthesis” and “lumazine binding domain” (**Figure 9A**), whereas at 3 h the uniquely upregulated DEGs were enriched in the “Defense response” biological process (**Figure 9B**). The common DEGs between the two time points showed enrichment in the biological process group of branched-chain amino acid biosynthetic process, cell adhesion, and amide biosynthetic process (**Figure 9C**). The list of significantly enriched GO terms, after GO analysis of DEGs associated with *S. aureus* during co-culturing with *S. anginosus* and tonsillar cells, is in **Supplementary Data Sheet S1**.

**Figure 10:**
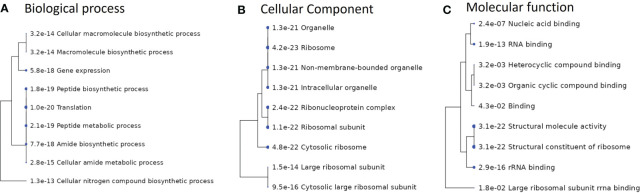
Enriched GO pathways of overlapping genes between *S. aureus* and *S. anginosus*. Hierarchical clustering trees summarize the correlation among pathways identified during enrichment analysis. The pathways with many shared genes are clustered together. A larger dot indicates a more significant P-value. **(A)** Top 10 enriched pathways involved in biological processes. **(B)** Top 10 enriched pathways involved in cellular component. **(C)** Top 10 enriched pathways involved in molecular functions.

In addition, a pathway enrichment analysis performed on the overlapping genes from *S. anginosus* and *S. aureus*, listed in **Supplementary Table S4** is also presented in **Figure 10**. The top 10 GO enriched pathways involved in biological process, cellular component and molecular functions were mainly found to be responsible in biosynthesis processes (**Figure 10A**), ribosomal subunits (**Figure 10B**) and RNA binding (**Figure 10C**), respectively. The GO enrichment analysis of overlapping genes (**Figure 10**) does not show the involvement of some of the important GO terms (such as cell adhesion, riboflavin biosynthesis and defense response), that were significantly enriched in *S. aureus* during co-culturing with *S. anginosus* (**Figure 9**). Overall, these findings provide insight into the involvement of the majority of biological processes in *S. aureus* during co-culturing together with evidence for enrichment in defense response only after 3 h of co-culturing with *S. anginosus* in the presence of tonsillar cells. Some of the enriched GO terms detected in this study using overlapping genes remain the same for both *S. aureus* and S. *anginosus.*

### 3.8 KEGG pathway analysis

**Figure 11:**
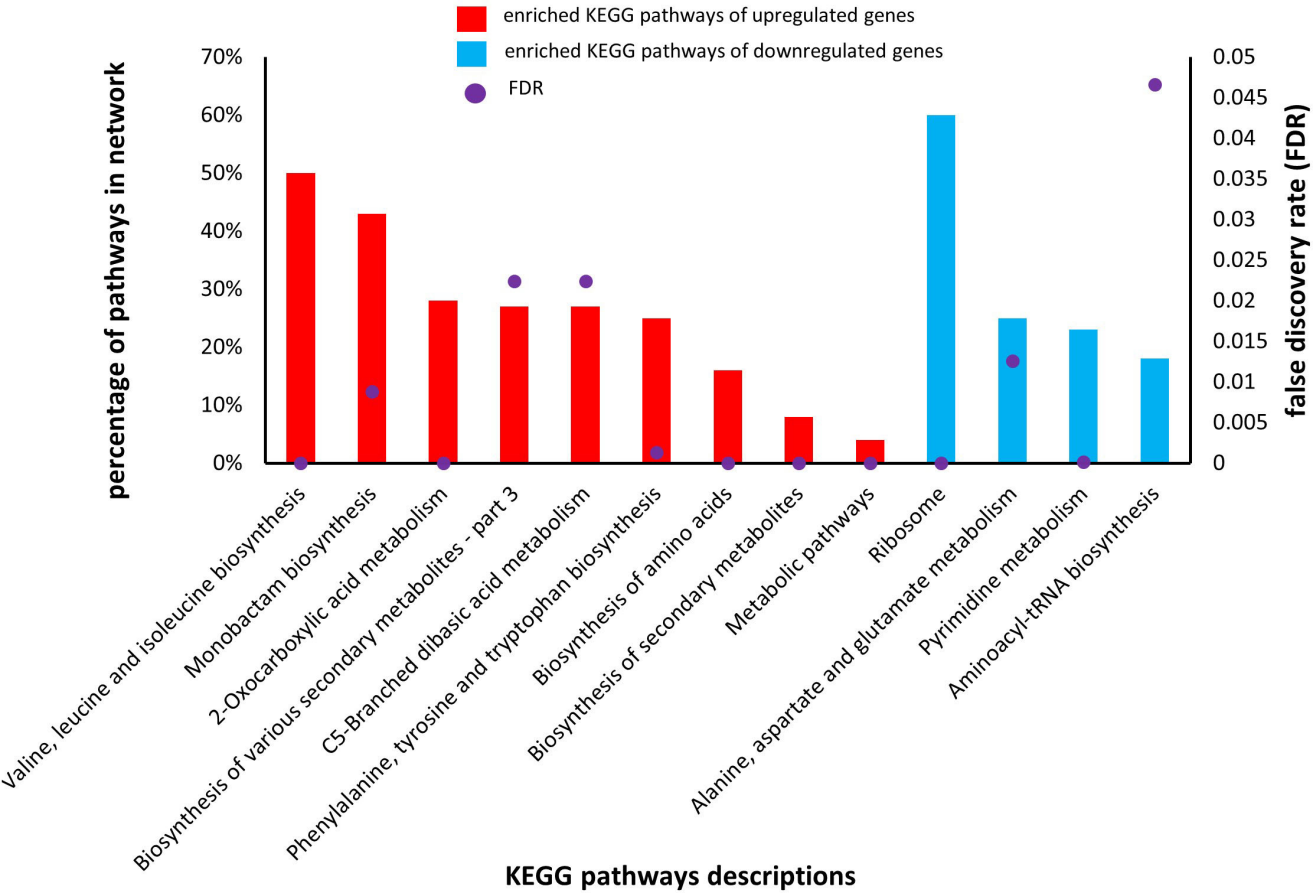
Enriched KEGG pathways of upregulated and downregulated genes in *S. aureus* co-culture with *S. anginosus* and tonsillar cells versus *S. aureus* co-cultured without tonsillar cells. Nine different KEGG pathways were associated with upregulated genes (red bars), whereas four different KEGG pathways were associated with downregulated genes (blue bars). Only significant pathways with a false discovery rate (FDR) < 0.05) are presented (purple dots).

KEGG analysis also uses DEGs involved in co-cultured *S. aureus* with *S. anginosus* in the presence of tonsillar cells compared to co-cultured *S. aureus* with *S. anginosus* without tonsillar cells, to identify the enriched pathways involved by DEGs. The KEGG pathway analysis revealed that the upregulated DEGs were significantly (FDR < 0.05) associated with pathways including “Valine, leucine and isoleucine biosynthesis”, “2- Oxocarboxylic acid metabolism”, “Phenylalanine, tyrosine and tryptophan biosynthesis”, “Biosynthesis of amino acids”, “Biosynthesis of secondary metabolites”, and “Metabolic pathways” (**Figure 11**). The percentage of enriched KEGG pathways identified from upregulated genes were ranging from 4 to 50% (**Figure 11**, red bar). The downregulated DEGs were significantly (FDR < 0.05) associated with “Ribosome” and “Pyrimidine metabolism” (**Figure 11**). The percentage of enriched KEGG pathways by downregulated genes were ranging from 15 to 60% (**Figure 11**, blue bar). Overall, the KEGG pathway analysis has provided valuable insights into the highly enriched significant pathways together with identification of ribosome shutdown by *S. aureus* during stress response.

### 3.9 Comparison of *S. aureus* DEGs upon mono- and co-culturing

**Figure 12:**
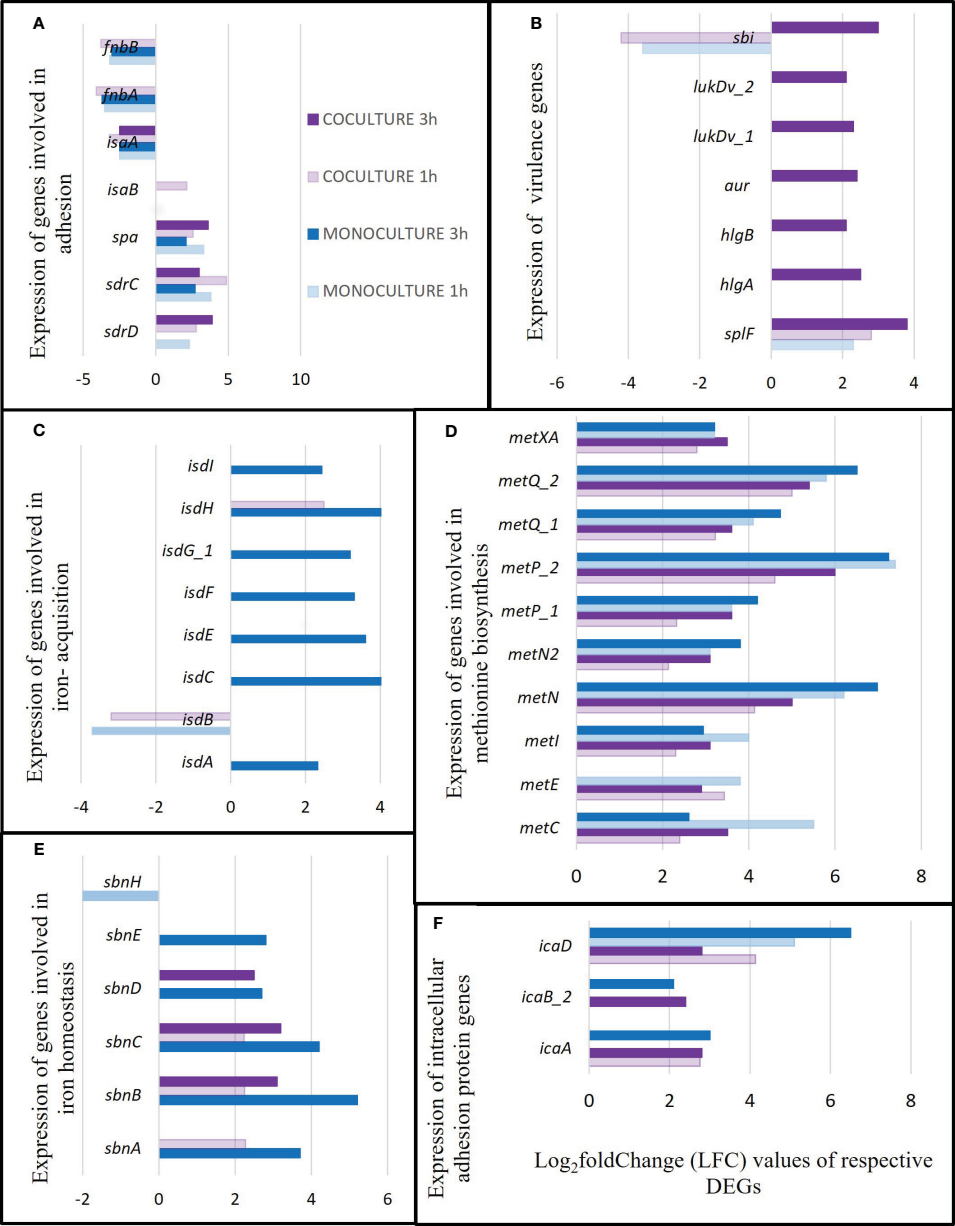
Several survival factors exhibited by *S. aureus* during co-culturing with *S. anginosus* and tonsillar cells compared to S. aureus monoculturing with tonsillar cells. The genes presented are the significant DEGs (p-value < 0.05 and log_2_FC ≥ |2|) identified from two separate datasets i.e., monoculturing was performed in a previous study and co-culturing in the present study. The dark and light purple color bars represent the DEGs from co-culture at 3 h and 1 hour, respectively, while the dark and light blue color bars represent the DEGs identified from monoculture at 3 h and 1 h, respectively. **(A)** Expression of genes encoding different adhesion factors. **(B)** Expression of virulence genes. **(C)** Expression of genes involved in iron-regulated surface genes. **(D)** Expression of genes involved in methionine biosynthesis. **(E)** Expression of genes involved in iron homeostasis. **(F)** Expression of genes involved in intracellular adhesion protein.

To gain a better understanding of the alteration of the transcriptome in *S. aureus* upon co-culturing, a broad comparison was made between the identified DEGs in *S. aureus* co-cultured with *S. anginosus* and tonsillar cells (present study, C_co_1/T_co_1 and C_co_3/T_co_3) versus DEGs in *S. aureus* with tonsillar cells (previous study, C1/T1 and C3/T3) as described in **Table 1**. The list of DEGs identified in *S. aureus* without *S. anginosus* were retrieved from a previous study (Bastakoti et al., 2023). Several *S. aureus* transcripts were identified, and a comparison of the DEGs identified from the present and previous study is presented in **Figure 12**; **Table 3**.

**Table 3:**
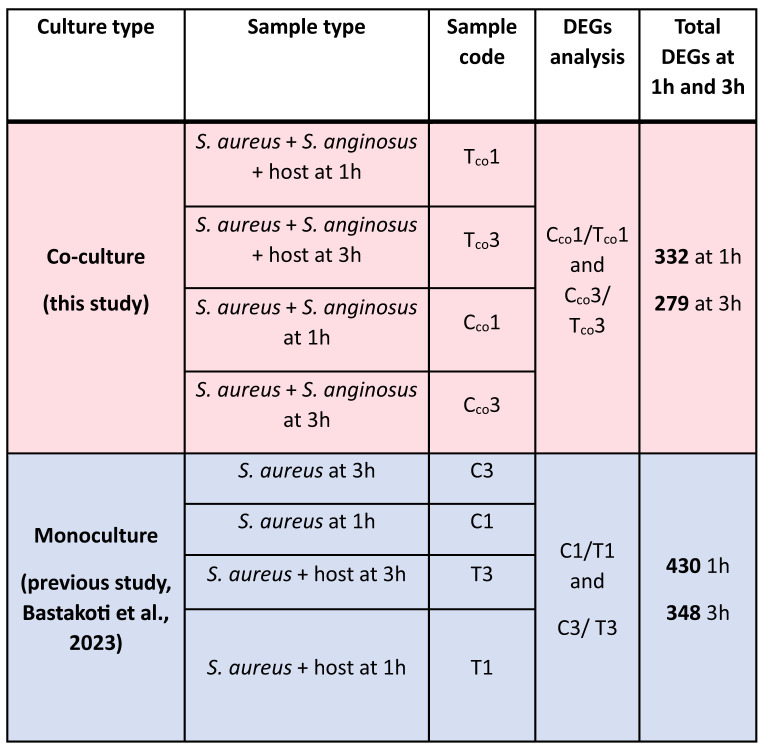
An overview of total DEGs in S. aureus influenced due to the presence of S. anginosus and tonsillar cells.

The red color shading represents the DEGs, 332 at 1h and 279 at 3h, identified in the present co-culture study. The blue color shading represents DEGs from a previous transcriptomics study.

Several DEGs were identified only in *S. aureus* (**Supplementary Table S4**). Most of the adhesion genes were differentially expressed in both monoculture (DEGs from our previous study) and co-culture condition (present study with *S. anginosus*). For instance, a significant downregulation of fibronectin-binding protein A (*fnbA*), fibronectin-binding protein B (*fnbB*), and staphylococcal antigen A (*isaA*) was seen with LFC ≤ -2 for all conditions except *fnbA* and *fnbB* in co-culture 3 h (LFC < -1.37, not presented in **Figure 12A**). There was an upregulation of staphylococcal protein A (*spa*) and serine-aspartate repeat-containing protein C (*sdrC*) regardless of culture conditions and time points. It appears that these genes are continuously differentially expressed in *S. aureus* when it meets tonsillar cells with or without *S. anginosus* (**Figure 12A**). *IsaB*, was upregulated only at 1 h of co-culturing, and *sdrD* was upregulated in both monoculture and co-culture after 1 h, but only after 3 h of co-culturing, being absent in 3 h monoculture. This indicates that *sdrD* expression is vital at 3 h of co-culturing with *S. anginosus*, compared to *S. aureus* alone, in the presence of host (**Figure 12A**).

Some of the genes encoding secreted toxins or enzymes were only expressed after 3 h of co-culturing of exposure to tonsillar cells, such as *lukD, aur*, *hlgA* and *hlgB* (**Figure 12B**). Most genes responsible for iron acquisition such as *isdA, isdB, isdC, isdE, isdF, isdG*, and *isdI* were not expressed in *S. aureus* during co-culturing with *S. anginosus* and host cells (**Figure 12C**). Expression of several genes involved in methionine biosynthesis was upregulated, with LFC ranging from 2 to 7, in all culturing conditions and time exposures (**Figure 12D**). Additionally, the genes responsible for iron homeostasis, *sbn*ABCDE, were found to be upregulated either in co-culture (1 h and 3 h) or in monoculture 3 h; however, *sbnH* was found to be downregulated only in monoculture after 1 h of exposure (**Figure 12E**). Some of the intercellular adhesion protein genes (*eg., icaA, icaB and icaD*) were also identified to be upregulated both in monoculture and co-culture in the presence of tonsillar cells (**Figure 12F**).

Similarly, the gene enrichment analysis of DEGs in *S. aureus* due to *S. anginosus* and tonsillar cells have revealed a small number of upregulated genes (15 genes, **Figure 8A** in x-axis) involved in GO terms in comparison to DEGs identified from monoculture study (25 genes (Bastakoti et al., 2023),). The pathways involved in “riboflavin biosynthesis” and “lumazine binding domain” were identified by upregulated genes only from 1 h datasets of co-culturing.

Taken together, the number of DEGs in *S. aureus* co-cultured with *S. anginosus* is found to be lower than DEGs identified in *S. aureus* without *S. anginosus*, both being analyzed in the presence of tonsillar cells. Nevertheless, several new sets of DEGs were identified when *S. aureus* was co-cultured, and some of the DEGs also present in *S. aureus* without being exposed to *S. anginosus* were also detected. This finding indicates that the presence of *S. anginosus* can influence the DEGs of *S. aureus* when exposed to tonsillar cells. Briefly, there are some core virulence factors exhibited by *S. aureus* that are expressed in every condition, and some are unique with respect to culturing condition and time point of exposure to host cells.

## 4 Discussion

A better understanding of the *S. aureus* throat colonization process together with other competing and/or coexisting microbes, may provide insight into *S. aureus* adaptation to throat and recurrence of colonization. In this work, we explored the responses triggered by the encounter of two common throat pathogens, *S. aureus* and *S. anginosus*, in the presence of human tonsil epithelial cells (HTEpiC). Previously, we verified the suitability of HTEpiC for studying the interaction between *S. aureus* and human tonsillar cells without compromising the viability of the host cells (Bastakoti et al., 2023). In this study, we aimed to identify the transcripts in *S. aureus* that are important when facing a potential competitor during throat colonization. We compared the transcriptome of *S. aureus* co-cultured with *S. anginosus* in the presence of tonsillar cells, and the transcriptome from *S. aureus* grown in monoculture (Bastakoti et al., 2023).

Several adhesion factors exhibited by *S. aureus* with/without *S. anginosus* in the presence/absence of tonsillar cells were identified. Many transcripts were differentially expressed in *S. aureus*, and these transcripts are likely to play an important role in *S. aureus* colonization in the presence of a competitor or they may be used by *S. aureus* to protect itself from competition. In contrast to a study by Hamamoto et al, which examined the virulent Newman strain to identify upregulated genes after infection in a mouse model (Hamamoto et al., 2022), our study focused on a strain colonizing the throat of a healthy individual.

The present co-culture study demonstrates the significant recovery of both bacteria, *S. aureus* and *S. anginosus*, when exposed to tonsillar cells. This is in contrast to another co-culture study which showed that *S. aureus* was outcompeted by *Pseudomonas aeruginosa* by producing inhibitory molecules (Filkins et al., 2015; Smith et al., 2017); however, the coexistence between these two pathogens could also be determined from the interactions between metabolism and growth (Woods et al., 2018; Price et al., 2020; Pajon et al., 2023). Thus, these studies indicate that certain bacterial strains can produce substances that give them a competitive advantage over other strains in a co-culture environment, but not necessarily kill the bacteria. The upregulation of several virulence genes during co-culture and expression of iron-regulatory genes mostly in *S. aureus* grown without *S. anginosus*, suggest that the presence of other bacteria, such as *S. anginosus*, could augment and affect the pathogenicity of *S. aureus*. Our observation is also consistent with the result of other co-culture studies with *S. aureus* and *P. aeruginosa* (Korgaonkar et al., 2013; Yang N. et al., 2020). Taken together, the presence of *S. anginosus* in the tonsillar cells could significantly impose a change in the transcriptomic level of *S. aureus* after co-culturing.

This study identified several DEGs in *S. aureus* when exposed to tonsillar cells and *S. anginosus* at 1 h and 3 h. Genes associated with the production of virulence factors, as well as genes involved in methionine biosynthesis, adhesion factors, iron-regulated surface genes, iron homeostasis genes, intercellular adhesion protein, defense response and other survival mechanisms were identified. Some of the genes encoding proteins involved in adhesion such as *isaB*, was upregulated in *S. aureus* at 1h of coculturing, and *sdrD*, which is commonly expressed after 1 h of monoculture and co-culture, was upregulated only after 3 h of co-culturing; virulence genes encoding secreted proteins were also highly upregulated; and iron-regulatory genes were not expressed in co-culture. This suggests that the presence of *S. anginosus* might create an environment in which *S. aureus* can better survive and express its virulence factors. Additionally, iron does not appear to be a limiting factor in the co-culturing environment as it is not upregulated. This suggests that *S. aureus* is able to adapt to changes in the environment by modulating the expression of specific genes in order to survive. In addition, the upregulation of virulence genes may suggest that *S. aureus* is able to use the resources from host or another bacterial species. Similarly, a previous co-culture transcriptomics study between *S. aureus* and *P. aeruginosa* indicated that *S. aureus* has a significant impact on the gene expression of genes involved in *P. aeruginosa* carbon and amino acid metabolism (Camus et al., 2020). A transcriptome study of *S. anginosus* when grown with *S. aureus* and *P. aeruginosa* in a biofilm has also shown the impact on the expression of genes involved in cell wall synthesis and on cell wall thickness (Tavernier et al., 2018).

Further, our transcriptome analysis of *S. aureus* co-cultured with *S. anginosus* and tonsillar cells indicated upregulation of genes involved in riboflavin biosynthesis and downregulation of Staphylococcal ribosomal protein-encoding genes. This contrasts with the recent finding from the transcriptional interplay between *S. aureus* and *Malassezia restricta* co-existing during skin colonization (Yang et al., 2023). Riboflavin is a precursor to essential co-enzymes, Flavin mononucleotide and flavin adenine dinucleotide (Fischer and Bacher, 2005). *S. aureus* generates riboflavin via *de novo* biosynthesis or obtains it from the host environment (Zhang et al., 2010; Gutiérrez-Preciado et al., 2015). It is possible that *S. aureus* and *S. anginosus* may compete for riboflavin uptake in the co-culture environment in the presence of host cells. The upregulation of genes involved in riboflavin biosynthesis might be one of the reasons for the increased growth of *S. aureus* (Yang et al., 2023).

The expression of iron acquisition genes in *S. aureus* during co-culturing with *S. anginosus* was not detected after being exposed to host cells. This expression was highly upregulated in the monoculture study, where *S. aureus* was not co-cultured with *S. anginosus* (Bastakoti et al., 2023). This is in contrast to other co-culture studies between *P. aeruginosa* and *S. aureus* where they detected that *P. aeruginosa* kills *S. aureus* to acquire iron using the LasA protease and disperses the *S. aureus* biofilm (Woods et al., 2018; Tognon et al., 2019). Thus, our study suggests that *S. aureus* may not be able to acquire iron from its environment during co-culturing. This indicates that *S. aureus* may acquire iron from other sources, or that iron is not a limiting factor in the co-culturing environment. However, further studies are needed to better understand the role of iron in the co-culturing of *S. aureus* and *S. anginosus* with tonsillar cells.

We identified alterations in the expression of various *S. aureus* survival factors (for instance adhesion, virulence, iron-acquisition, and iron homeostasis genes) upon mono and co-culturing with *S. anginosus* and tonsillar cells. To identify the transcripts that were affected due to presence of *S. anginosus*, we compared survival factors exhibited by *S. aureus* during co-culturing with *S. anginosus* in the presence of tonsillar cells with our previous study where *S. aureus* alone was exposed to tonsillar cells (Bastakoti et al., 2023). Most of the DEGs were common to monoculture but the expression level varied, and some genes were uniquely up/down regulated only in presence of *S. anginosus*. Interestingly, *isaB* was only upregulated in co-culture (1 h) with *S. anginosus* and tonsillar cells, and *sdrD* was upregulated at all conditions, except in *S. aureus* grown alone (3 h). Furthermore, some of the genes involved in secretion of toxins, such as *lukD*, *aur, hlgA* and *hlgB* were also expressed only after 3 h of co-culturing with *S. anginosus* and tonsillar cells. The results indicate that the expression of adhesins and toxins is not simultaneous, but rather follows a precise temporal sequence. It is therefore likely that adhesins play an important role in the attachment of *S. aureus* to tonsillar cells, followed by the secretion of toxins for further infection. In line with this, a previous study has indicated that interaction between *S. aureus* and *P. aeruginosa* can be beneficial for colonization and further lead toward pathogenicity (Alves et al., 2018). Our results are similar to the results of a previous study performed on *S. aureus* and *P. aeruginosa*, where the presence of *P. aeruginosa* caused changes in the transcriptome of *S. aureus* during internalization into epithelial cells (Briaud et al., 2019). This suggests that the presence of different microbes can have a significant effect on the transcriptome of *S. aureus* and could potentially have significant implications for the spread and control of bacterial infections.

We have also identified a set of DEGs in *S. aureus* that are involved in various pathways, such as amino acid metabolism, biosynthesis of secondary metabolites, biological adhesion, ribosomal protein, and rRNA binding, which all play a key role in ensuring the proper functioning and regulation of the biological system. Each of these processes and pathways might play a critical role in making *S. aureus* as a potential colonizer to the host cells (Schoenfelder et al., 2013; Alreshidi et al., 2022; Samuel et al., 2022). For instance, secondary metabolites are essential for producing molecules that enable *S. aureus* to adhere to host cells and cause infection. Importantly, our study has shown that certain genes exhibited by *S. aureus* play a key role in the response to *S. anginosus* while meeting tonsillar cells. The number of genes involved in GO terms during co-culturing were found to be less than monoculture, indicating that some sets of genes are not necessary to be differentially expressed during co-culturing with *S. anginosus*. Nevertheless, there were some GO terms such as “riboflavin biosynthesis”, “lumazine binding domain” and “defense response”, which were identified to be upregulated only during co-culturing environment. Thus, these identified genes, involved in several GO terms, are of particular importance for *S. aureus* and may be essential for its survival and adaptation to changing environments. It is important to note that there are overlapping genes in *S. aureus* and *S. anginosus* and this was expected. Some of the potential overlapping DEGs between *S. aureus* and *S. anginosus* were also found to be significantly enriched in GO terms related to ribosomal subunits and RNA binding. These enriched GO terms suggest that the same genes contribute to the assembly and functioning of ribosomes, as well as the regulation of RNA metabolism in both *S. aureus* and *S. anginosus.* In general, our findings demonstrate the importance of these genes in responding to *S. anginosus* in the presence of tonsillar cells and may lead to new treatments of infections or other diseases associated with throat colonization by *S. aureus.*

There are some limitations to this study: (1) Current results could change if different strains were used in the experiments because of intra-strain variability of expression landscape and colonization ability in *S. aureus*. We know that some strains are better adapted to humans, but repeated host adaptation events have happened in both human and animal directions (Richardson et al., 2018). (2) The transcriptomics profiling is performed only for *S. aureus* but neither for *S. anginosus* nor the host cell. The investigation of DEGs in *S. anginosus* might have revealed the unique and common genes involved by each bacterium during exposure to tonsillar cells (3). The number of reads mapped against the *S. anginosus* reference genome was very low, which could have resulted due to low recovery of *S. anginosus* RNA during total RNA extraction from co-culture samples. In line with this, a previous transcriptomic study of *S. anginosus* growing in a multispecies biofilm has also indicated a lower proportion of mapped reads against *S. anginosus* (Tavernier et al., 2018). In the case of RNA-seq for the *S. anginosus* mixed sample, it might be appropriate to increase the sequencing depth, so that the number of reads and aligned reads can be increased. Moreover, in our study, the separate sample clustering observed in the PCA plot also indicates the clear variation and gene clustering. Additionally, some of the significant DEGs variations observed in co-culture compared to monoculture, indicates that the presence of a low number of *S. anginosus* reads is enough to identify transcriptomics alteration in *S. aureus* during co-culturing.

In conclusion, our study identified several transcripts in *S. aureus* that might be important when facing a potential competitor during throat colonization. Alterations in expression of various *S. aureus* survival factors were observed when co-cultured with *S. anginosus* and tonsillar cells, especially in genes encoding adhesion protein, secreted proteins, and iron-acquisitions. These findings may be useful in the development of interventions against *S. aureus* throat colonization and suggest that a further investigation of the expression landscape is warranted to gain an improved understanding of the role of co-colonization in the host immune response.

## Data availability statement

The datasets presented in this study can be found in online repositories. The names of the repository/repositories and accession number(s) can be found below: https://www.ncbi.nlm.nih.gov/geo/, GSE234900; https://www.ebi.ac.uk/ena, PRJEB59355.

## Ethics statement

Ethical approval was not required for the studies on humans in accordance with the local legislation and institutional requirements because only commercially available established cell lines were used.

## Author contributions

SB: Writing – original draft, Writing – review & editing, Data curation, Formal analysis, Investigation, Methodology, Validation, Visualization. MP: Formal analysis, Validation, Writing – review & editing, Visualization. CA: Supervision, Writing – review & editing. KJ: Writing – review & editing, Methodology. JC: Writing – review & editing. MJ: Conceptualization, Funding acquisition, Project administration, Resources, Supervision, Validation, Writing – review & editing, Visualization. A-MH: Conceptualization, Funding acquisition, Project administration, Resources, Supervision, Validation, Writing – review & editing, Visualization.
