# Supplementary material for: Co-culturing with Streptococcus anginosus alters Staphylococcus aureus transcriptome when exposed to tonsillar cells
Source: Front Cell Infect Microbiol. 2024 Jan 25;14:1326730. doi: 10.3389/fcimb.2024.1326730 (PMC10850355; doi:10.3389/fcimb.2024.1326730)
Supplement: Supplementary file 1 [file DataSheet_1.pdf]

Data Sheet 1: Gene ontology analysis of differentially expressed genes associated with *S. aureus* during coculturing with *S. anginosus* and tonsillar cells.

Gene name	Term/gene function	Gene count	Enrichment FDR
SBNA, METE, TRPC, TRPF, TRPB, DAPA, DAPB, ILVD, ILVC, LEUB, LEUC, LEUD, ILVA	GO:1901607/Alpha-amino acid biosynthetic process	13	< 0.05
SBNA, METE, TRPC, TRPF, TRPB, DAPA, DAPB, ILVD, ILVC, LEUB, LEUC, LEUD, ILVA	GO:0008652/Cellular amino acid biosynthetic process	13	< 0.05
ILVD, ILVC, LEUB, LEUC, LEUD, ILVA	GO:0009081/Branched-chain amino acid metabolic process	6	< 0.05
ILVD, ILVC, LEUB, LEUC, LEUD, ILVA	GO:0009082/Branched-chain amino acid biosynthetic process	6	< 0.05
HUTH, SBNA, METE, TRPC, TRPF, TRPB, DAPA, DAPB, ILVD, ILVC, LEUB, LEUC, LEUD, ILVA	GO:1901605/Alpha-amino acid metabolic process	14	< 0.05
SBNA, METE, TRPC, TRPF, TRPB, DAPA, DAPB, ILVD, ILVC, LEUB, LEUC, LEUD, ILVA	GO:0046394/Carboxylic acid biosynthetic process	13	< 0.05
SBNA, METE, TRPC, TRPF, TRPB, DAPA, DAPB, ILVD, ILVC, LEUB, LEUC, LEUD, ILVA	GO:0016053/Organic acid biosynthetic process	13	< 0.05
HUTH, SBNA, METE, TRPC, TRPF, TRPB, DAPA, DAPB, ILVD, ILVC, LEUB, LEUC, LEUD, ILVA	GO:0006520/Cellular amino acid metabolic process	14	< 0.05
HUTH, SBNA, TRPC, TRPB, DAPA, ILVD, LEUC, LEUD, ILVA	GO:0016829/Lyase activity	9	< 0.05
ILVD, ILVC, ILVA	GO:0006549/Isoleucine metabolic process	3	< 0.05
LEUB, LEUC, LEUD	GO:0006551/Leucine metabolic process	3	< 0.05
ILVD, ILVC, ILVA	GO:0009097/Isoleucine biosynthetic process	3	< 0.05
LEUB, LEUC, LEUD	GO:0009098/Leucine biosynthetic process	3	< 0.05
ESXA, SLE1, SDRC, SDRD, ISDH, SPLF, SPLE, SBI, HLGB	GO:0005576/Extracellular region	9	< 0.05

SBNA, METE, TRPC, TRPF, TRPB, DAPA, DAPB, RIBH, ILVD, ILVC, LEUB, LEUC, LEUD, ILVA	GO:0044283/Small molecule biosynthetic process	14	< 0.05
TRPB, DAPA, ILVD, LEUC, LEUD	GO:0016836/Hydro-lyase activity	5	< 0.05
SDRC, SDRD, ICAA	GO:0007155/Cell adhesion	3	< 0.05
SDRC, SDRD, ICAA	GO:0022610/Biological adhesion	3	< 0.05
SBNA, TRPB, ILVA	IPR001926/Pyridoxal-phosphate dependent enzyme	3	< 0.05
SBNA, TRPB, ILVA	IPR036052/Tryptophan synthase beta subunit-like PLP-dependent enzyme	3	< 0.05
HUTH, SBNA, METE, TRPC, TRPF, TRPB, DAPA, DAPB, ILVD, ILVC, LEUB, LEUC, LEUD, ILVA	GO:0019752/Carboxylic acid metabolic process	14	< 0.05
HUTH, SBNA, METE, TRPC, TRPF, TRPB, DAPA, DAPB, ILVD, ILVC, LEUB, LEUC, LEUD, ILVA	GO:0043436/Oxoacid metabolic process	14	< 0.05
TRPB, DAPA, ILVD, LEUC, LEUD	GO:0016835/Carbon-oxygen lyase activity	5	< 0.05
METE, DAPA, DAPB, ILVA	GO:0009066/Aspartate family amino acid metabolic process	4	< 0.05
TRPC, TRPF, TRPB	GO:0000162/Tryptophan biosynthetic process	3	< 0.05
TRPC, TRPF, TRPB	GO:0006568/Tryptophan metabolic process	3	< 0.05
TRPC, TRPF, TRPB	GO:0006586/Indolalkylamine metabolic process	3	< 0.05
TRPC, TRPF, TRPB	GO:0042430/Indole-containing compound metabolic process	3	< 0.05
TRPC, TRPF, TRPB	GO:0042435/Indole-containing compound biosynthetic process	3	< 0.05
TRPC, TRPF, TRPB	GO:0046219/Indolalkylamine biosynthetic process	3	< 0.05
SDRC, SDRD, ISDH	PF04650/YSIRK type signal peptide	3	< 0.05
METE, DAPA, DAPB	GO:0009067/Aspartate family amino acid biosynthetic process	3	< 0.05
SDRC, SDRD, ISDH	IPR019931/LPXTG cell wall anchor domain	3	< 0.05

DAPA, DAPB	GO:0019877/Diaminopimelate biosynthetic process	2	< 0.05
SDRC, SDRD	PR013783/Immunoglobulin-like fold	2	< 0.05
SDRC, SDRD	PF13620/Carboxypeptidase regulatory-like domain	2	< 0.05
SDRC, SDRD	PF17210/SdrD B-like domain	2	< 0.05
HUTH, SBNA, METE, TRPC, TRPF, TRPB, DAPA, DAPB, ILVD, ILVC, LEUB, LEUC, LEUD, ILVA	GO:0006082/Organic acid metabolic process	14	< 0.05
SDRC, SDRD, ISDH	GO:0005618/Cell wall	3	< 0.05
TRPC, TRPF, TRPB	GO:0006576/Cellular biogenic amine metabolic process	3	< 0.05
TRPC, TRPF, TRPB	GO:0009308/Amine metabolic process	3	< 0.05
TRPC, TRPF, TRPB	GO:0009309/Amine biosynthetic process	3	< 0.05
SDRC, SDRD, ISDH	GO:0030312/External encapsulating structure	3	< 0.05
TRPC, TRPF, TRPB	GO:0042401/Cellular biogenic amine biosynthetic process	3	< 0.05
TRPC, TRPF, TRPB	GO:0044106/Cellular amine metabolic process	3	< 0.05
ESXA, SLE1, SPLF, SPLE, HLGB	GO:0005576/Extracellular region	5	< 0.05
RIBH, RIBBA	Riboflavin biosynthesis, and Lumazine binding domain	2	< 0.05
SLE1, HLGB	GO:0019835/Cytolysis	2	< 0.05
SLE1	GO:0042742/Defense response to bacterium	1	< 0.05
RPSF, RPSR, RPLK, RPLA, RPLJ, RPLL, RPSG, RPLS, RPSB, RPMA, RPLT, RPMI, RPSI, RPLM, RPSE, RPLR, RPLF, RPSH, RPLE, RPLX, RPLN, RPSQ, RPMC, RPLP, RPSC, RPLV, RPSS, RPLB, RPLW, RPLC	GO:0022626/Cytosolic ribosome	30	< 0.05

RPSF, RPSR, RPLK, RPLA, RPLJ, RPLL, RPSL, RPSG, RPSP, RIMM, RPLS, RPSB, RPMA, RPLU, RPLT, RPMI, RPSD, RPSI, RPLM, RPSE, RPLR, RPLF, RPSH, RPLE, RPLX, RPLN, RPSQ, RPMC, RPLP, RPSC, RPLV, RPSS, RPLB, RPLW, RPLD, RPLC	GO:0043226/Organelle	36	< 0.05
RPSF, RPSR, RPLK, RPLA, RPLJ, RPLL, RPOB, RPOC, RPSL, RPSG, FUSA, PHES, ILES, PYRR, RPSP, RIMM, TRMD, RPLS, RPSB, FRR, RIMP, INFB, ASPS, HISS, RPMA, RPLU, RPLT, RPMI, INFC, RPSD, TYRS, PRFA, RPSI, RPLM, RPSE, RPLR, RPLF, RPSH, RPLE, RPLX, RPLN, RPSQ, RPMC, RPLP, RPSC, RPLV, RPSS, RPLB, RPLW, RPLD, RPLC	GO:0010467/Gene expression	51	< 0.05
RPLK, RPLA, RPLJ, RPLL, RPLS, RPMA, RPLT, RPMI, RPLM, RPLR, RPLF, RPLE, RPLX, RPLN, RPMC, RPLP, RPLV, RPLB, RPLW, RPLC	GO:0015934/Large ribosomal subunit	20	< 0.05
RPSF, RPSR, RPSL, RPSG, RPSP, RPSB, RPSD, RPSI, RPSE, RPSH, RPSQ, RPSC, RPSS	GO:0015935/Small ribosomal subunit	13	< 0.05
RPSF, RPSR, PDXS, PDXT, RPLK, RPLA, RPLJ, RPLL, RPOB, RPOC, RPSL, RPSG, FUSA, ARGG, PHES, ILES, PYRR, PYRB, CARB, PYRF, RPSP, RIMM, TRMD, RPLS, RPSB, PYRH, FRR, RIMP, INFB, GUAC, ASPS, HISS, RPMA, RPLU, RPLT, RPMI, INFC, RPSD, TYRS, CSHA, PRFA, PYRG, RPSI, RPLM, RPSE, RPLR, RPLF, RPSH, RPLE, RPLX, RPLN, RPSQ, RPMC, RPLP, RPSC, RPLV, RPSS, RPLB, RPLW, RPLD, RPLC	GO:0034641/Cellular nitrogen compound metabolic process	61	< 0.05
CNTF, CNTD, CNTC, CNTB, CNTA	GO:0006824/Cobalt ion transport	5	< 0.05
CNTF, CNTD, CNTC, CNTB, CNTA	GO:0070838/Divalent metal ion transport	5	< 0.05
