# Supplementary material for: Co-culturing with Streptococcus anginosus alters Staphylococcus aureus transcriptome when exposed to tonsillar cells
Source: Front Cell Infect Microbiol. 2024 Jan 25;14:1326730. doi: 10.3389/fcimb.2024.1326730 (PMC10850355; doi:10.3389/fcimb.2024.1326730)
Supplement: Supplementary file 2 [file Image_1.pdf]

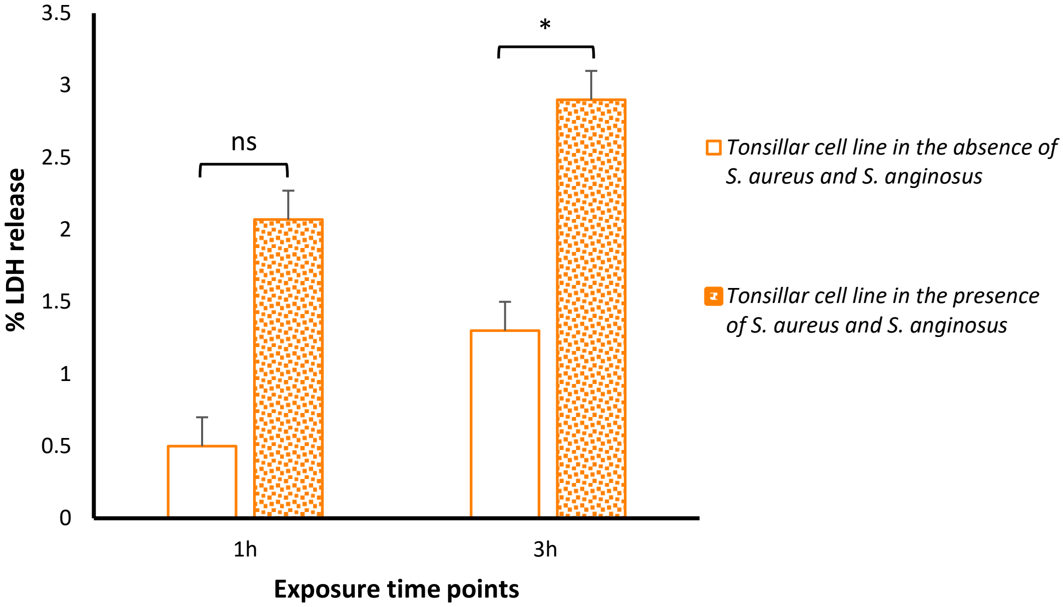

Figure S1. Co-culturing of S. aureus and S. anginosus shows minor cytotoxicity to the tonsillar cell line. *LDH release from tonsillar cells into the supernatant was measured following exposure with/without S. aureus and S. anginosus for 1 h and 3 h. The orange bar without dot represents the percentage (%) of LDH released by the tonsillar cell line in the absence of bacterial coculture (negative control) whereas dotted orange bar presents the LDH % release by the tonsillar cell line in the presence of bacterial co-culture. The bacterial cytotoxicity was calculated as a percentage of maximum LDH release control (positive control). The results are based on three independent experiments.*
