# Supplementary material for: Co-culturing with Streptococcus anginosus alters Staphylococcus aureus transcriptome when exposed to tonsillar cells
Source: Front Cell Infect Microbiol. 2024 Jan 25;14:1326730. doi: 10.3389/fcimb.2024.1326730 (PMC10850355; doi:10.3389/fcimb.2024.1326730)
Supplement: Supplementary file 3 [file Image_2.pdf]

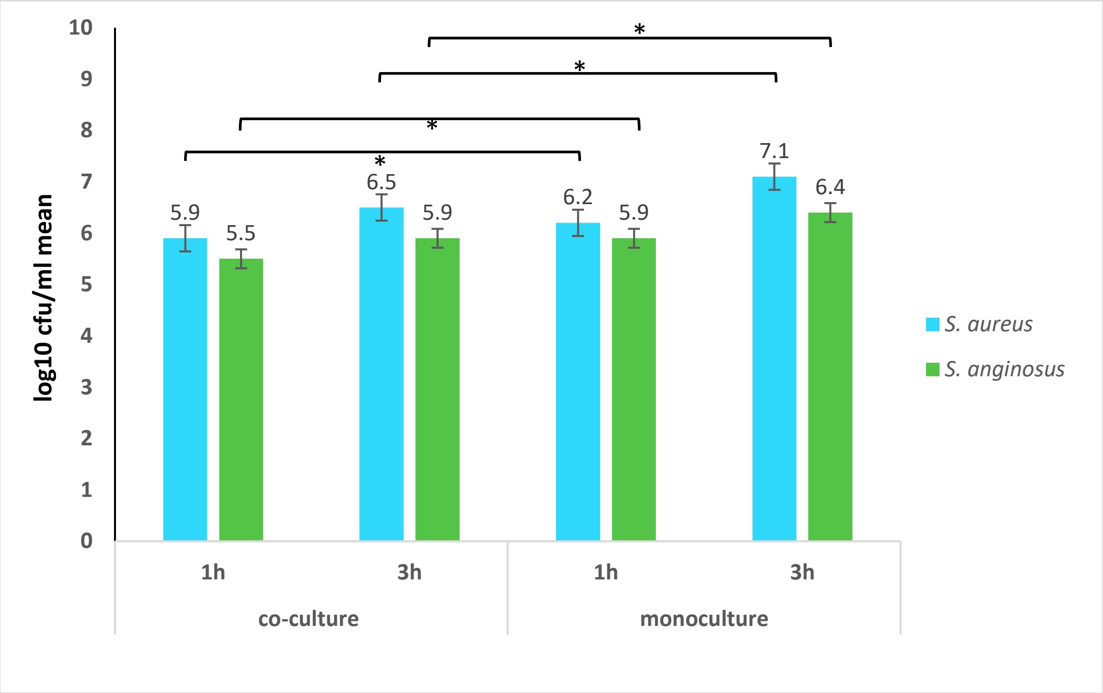

Figure S2. S. aureus and S. anginosus grown in the co-cultures and monocultures for 1 or 3 hours in the absence of host cells. *S. aureus and S. anginosus when grown alone is referred to as monoculture, and when grown together is referred to as co-cultured, both in tonsillar cell media for 1 and 3 hours, before being plated on selected media for CFU enumeration. The results are presented as mean log10 CFU/ml from three independent experiments. Error bars represent the +/- SD. Differences in the means between the groups were tested using a two-sample Student t-test. *P < 0.05.*
