# Supplementary material for: Co-culturing with Streptococcus anginosus alters Staphylococcus aureus transcriptome when exposed to tonsillar cells
Source: Front Cell Infect Microbiol. 2024 Jan 25;14:1326730. doi: 10.3389/fcimb.2024.1326730 (PMC10850355; doi:10.3389/fcimb.2024.1326730)
Supplement: Supplementary file 4 [file Image_3.pdf]

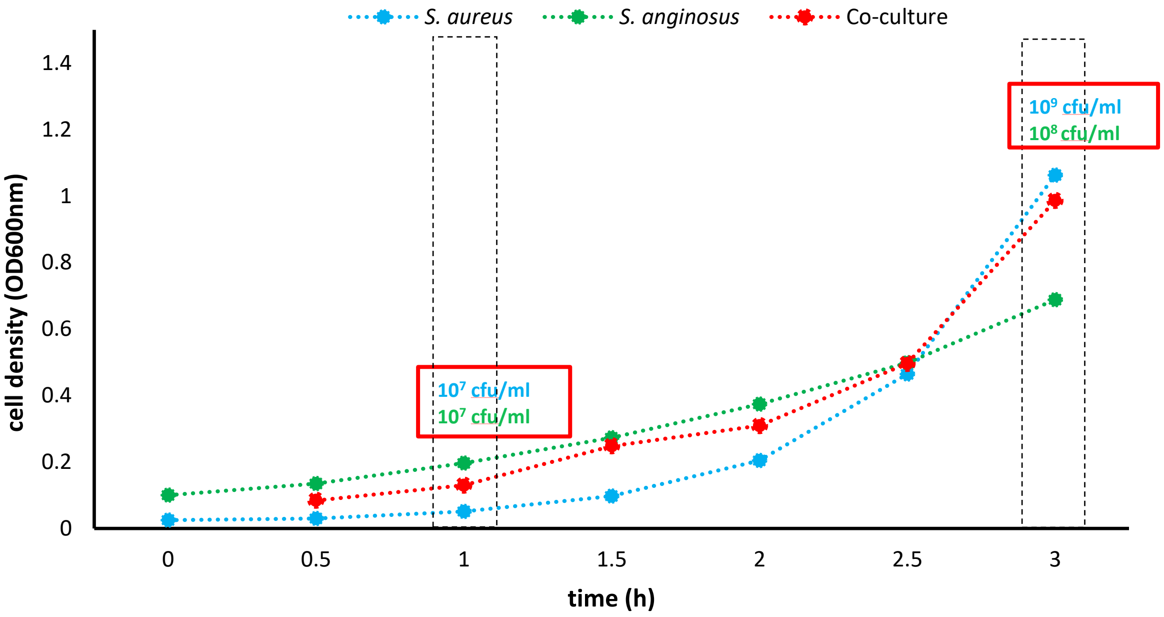

Figure S3. S. aureus and S. anginosus growth in bacteriological media. *Both the bacteria were grown on BHI overnight at 37 °C, and then the fresh bacterial culture was prepared (1:10) for monoculture (single bacterial strain growth) and co-culturing (S. aureus grown in the presence of S. anginosus). Cell density (OD600 nm) was measured every 30 mins for up to 3 hours (h). After 1 h and 3 h (marked with bars) bacteria were plated for colony-forming units (CFU) enumeration. CFU/ml of respective strains during co-culturing is indicated inside the red boxes, which is similar to enumeration from monoculture. Plate enumeration for monoculture samples was performed in BHI plate whereas selective media was used for co-cultured samples, such as CHROMagar™ for S. aureus counting and COBA plate for S. anginosus. The results show one representative experiment from a triplicate.*
